# Consonant and Vowel Processing in Word Form Segmentation: An Infant ERP Study

**DOI:** 10.3390/brainsci8020024

**Published:** 2018-01-31

**Authors:** Katie Von Holzen, Leo-Lyuki Nishibayashi, Thierry Nazzi

**Affiliations:** 1 Laboratoire Psychologie de la Perception, CNRS–Université Paris Descartes, 75006 Paris, France; ll.nishibayashi@gmail.com; 2 Department of Hearing and Speech Sciences, University of Maryland, College Park, MD 20740, USA; 3 Laboratory for Language Development, Riken Brain Science Institute, Wako-shi, Saitama-ken 351-0198, Japan

**Keywords:** lexical processing, word form segmentation, consonant bias, individual variability, Event-related brain potentials (ERPs), French-learning infants

## Abstract

Segmentation skill and the preferential processing of consonants (C-bias) develop during the second half of the first year of life and it has been proposed that these facilitate language acquisition. We used Event-related brain potentials (ERPs) to investigate the neural bases of early word form segmentation, and of the early processing of onset consonants, medial vowels, and coda consonants, exploring how differences in these early skills might be related to later language outcomes. Our results with French-learning eight-month-old infants primarily support previous studies that found that the word familiarity effect in segmentation is developing from a positive to a negative polarity at this age. Although as a group infants exhibited an anterior-localized negative effect, inspection of individual results revealed that a majority of infants showed a negative-going response (Negative Responders), while a minority showed a positive-going response (Positive Responders). Furthermore, all infants demonstrated sensitivity to onset consonant mispronunciations, while Negative Responders demonstrated a lack of sensitivity to vowel mispronunciations, a developmental pattern similar to previous literature. Responses to coda consonant mispronunciations revealed neither sensitivity nor lack of sensitivity. We found that infants showing a more mature, negative response to newly segmented words compared to control words (evaluating segmentation skill) and mispronunciations (evaluating phonological processing) at test also had greater growth in word production over the second year of life than infants showing a more positive response. These results establish a relationship between early segmentation skills and phonological processing (not modulated by the type of mispronunciation) and later lexical skills.

## 1. Introduction

To begin learning words, infants must first find and extract the word forms present in speech. For infants to later link these word forms to concepts, they must also form a phonologically detailed representation of these word forms. In the current study, we focus on how infants extract or *segment* word forms from the speech stream and subsequently process the phonological detail of these newly segmented words. Jusczyk and Aslin [1] were the first to reveal word segmentation abilities in young, i.e., 7.5-month-old, American English-learning infants. Following this seminal study, two decades of research have extended word segmentation abilities to infants learning a variety of languages (Dutch, Japanese, Spanish, German) [2,3,4,5,6], including French [6,7,8]. Importantly, behavioral studies [9] and studies using Event-related potentials (ERPs) [10,11] have established a link between early word segmentation skills and language outcomes later in development, which suggests that early segmentation abilities play a role in lexical acquisition. During that same developmental period, infants are learning the phonological properties of their native language [12], which may impact the way they process the phonological details of the word forms they segment. For French, recent evidence has found a shift between six and eight months from preferentially processing vowel information [13,14] to preferentially processing consonant information (C-bias) [13] in lexical processing. This C-bias is found in adults in many languages [15] and it has been proposed that its emergence bootstraps the acquisition of words [16], although no data so far supports this hypothesis. In the present study, we used ERPs to explore the neural basis of early segmentation abilities and of phonological abilities implicated in the processing of newly segmented word forms (exploring potential differences between consonant and vowel processing), focusing on French-learning infants. For both of these abilities, we explored whether they relate to later lexical abilities.

During the second half of their first year, ERP studies show that infants are maturing in their segmentation skills, as revealed by developmental changes in the polarity of the response to a segmented word (from positive to negative). Kooijman and colleagues [4] first examined word segmentation using ERPs with 10-month-old infants learning Dutch, finding a more negative response over left anterior electrodes to familiarized target words compared to novel control words when both were embedded in the speech stream (see also: [7,17]). When seven-month-old Dutch-learning infants were tested on the same set of stimuli, however, their response to familiarized target words was more positive compared to novel control words [10]. Männel and Friederici [2] tested German-learning infants and also found a shift from a positive response at six months, to a negative response at nine and 12 months. This polarity change has been attributed to increasing linguistic experience and cortex maturation over the first year of life. Although there were some small variations in timing and distribution of the effect, studies using ERPs to investigate early segmentation skills have found a difference in response to familiarized target and control words between 200 and 650 ms over anterior and/or left electrode sites, shifting from a positivity to a negativity between six and nine months (for a review see [18], Table 1 page 181). 

In addition to changes in polarity over development, variation in the polarity of word segmentation response has also been found within groups of similar-aged infants. Kooijman et al. [10] (see also [11,18]) found that overall, seven-month-old Dutch-learning infants’ response to familiarized target words was more positive in the right anterior region and more negative in the left posterior region than the response to novel control words. Correlation analyses revealed that the separate overall effects in right anterior and left posterior regions were driven by different subsets of infants, whose differing response polarities subsequently cancelled out effects in the typical anterior left region. Infants were thus separated into two subgroups: Negative Responders who showed the expected left anterior negative response to familiarized target words and Positive Responders who showed a positive response over left anterior electrodes to familiarized target words. By examining response polarity over left anterior electrodes, Kooijman and colleagues [10] were able to identify those younger infants who had already developed a negative response that is typically localized to this area in older infants [2,4,11,18]. Importantly, Negative Responders had better language skills later in development than Positive Responders (see also [11]). For older, 10-month-old infants, Negative Responders also showed an increasingly negative response to target word tokens embedded in passages in the Familiarization Phase, whereas Positive Responders did not [18]. Although these developmental changes suggest that infants eventually develop an anterior left word segmentation response with a negative polarity, those infants who develop the negative response earlier have more mature segmentation skills which it has been suggested are linked to more mature cortical development [2] and ultimately greater linguistic skills later in development [10,11].

Based on these findings, we extended previous evidence of segmentation using ERPs with French-learning 10-month-olds [7] to examine variability in segmentation in younger, eight-month-old French-learning infants. Using a passage–word paradigm similar to Männel and Friederici [2], we record eight-month-old French-learning infants’ ERP responses to CVC (i.e., consonant-vowel-consonant) target words (e.g., cave /kav/) embedded in eight sentences (Familiarization Phase) and isolated tokens of the target and unfamiliar control words (Test Phase). In the Familiarization Phase, a difference in response between the initial (1st and 2nd) and final (7th and 8th) repetitions of the target words is considered as an indication that infants are segmenting the target words from the speech stream. This change in response with increasing repetitions is thought to reflect memory trace formation, as infants begin to form a lexical entry for the segmented target word. Nine- to 12-month-olds [2,18] show a more negative response over left anterior electrodes for target words presented later versus earlier in the Familiarization Phase. No difference was found, however, for six-month-old infants [2], suggesting that this response is not yet established or more difficult to reveal in younger infants. Our study will examine whether at eight months infants exhibit evidence of memory trace formation during a segmentation task. 

Following each Familiarization Phase, we presented infants with isolated word tokens in a Test Phase. A difference in ERP response between target and phonologically unrelated, novel control words is considered a familiarity response, indicating that infants have segmented the target words in the Familiarization Phase. At the group level, Männel and Friederici [2] found that six-month-olds exhibit a positive familiarity response, but only when the target words were accentuated in the Familiarization Phase, while nine-month-olds show an overall negative familiarity response. In our study, ERP responses to familiarized target words (e.g., cave) were compared to phonologically unrelated CVC control words (e.g., reg (/rεg/). At eight months, infants may be transitioning from the early positive to the later negative familiarity response, making it difficult to predict results at the group level. However, we expected individual variation in the Test Phase familiarity response [10,18], with one subgroup of infants exhibiting more negative responses to target compared to control words (Negative Responders) and another subgroup of infants exhibiting more positive responses (Positive Responders). 

To examine whether response polarity is related to later achievement in vocabulary development, we explored whether it predicts vocabulary growth during the second year of life. Similar to Tsao et al. [19], we measure vocabulary at test age (eight months) as well as three follow-up ages: 13, 16, and 24 months. Infants begin to produce their first words at 13 months [20], have on average a productive vocabulary of 50 words at 16 months [21], and can combine words by 24 months [22]. Unlike Tsao and colleagues, however, we examined vocabulary growth using a growth curve analysis [23,24] to capture the relationship between response polarity and overall vocabulary achievement as well as the shape of change over time across multiple ages. 

While infants’ segmentation skills are changing, their processing of phonological information is also changing, which could impact how they represent and process word forms. For example, studies on Italian- and French-learning infants showed that an early bias for vowels in lexical processing at five–six months [13,14,25] becomes a C-bias at eight–eleven months [13,26,27]. Note that these biases do not necessarily mean that infants are not sensitive to both consonant and vowel information, but rather that when compared to one another, infants give more *weight* to one kind of phonological information, which is often referred to as a preference. By using ERPs we can examine responses separately, allowing an investigation of the phonological detail of newly segmented word processing, and whether this processing differs for consonants and vowels.

Together with studies showing that infants learning other languages (e.g., English, Danish) show a different developmental trajectory, the developmental changes found in French and Italian suggest that the C-bias is learned rather than innate, and might be related to the acquisition of the lexical [28] or acoustic/phonetic [29] regularities of the native language (for a review of the developmental origins of the C-bias, see [15]). Therefore, infants who learn to preferentially process consonants earlier may have an advantage in lexical development over infants who develop this preference later. In terms of the potential link between the two abilities explored in the present study, emergence of a C-bias may help the infant more efficiently segment the speech stream or may be a resulting effect of an infant who has developed more mature segmentation skills.

At eight months, French-learning infants have been found in a behavioral task to preferentially process consonants over vowels in word form segmentation, providing the earliest evidence of a C-bias [13]. These conclusions about preferences and biases come from comparing onset consonant mispronunciations to either control words or vowel mispronunciations (though not to target words), and from comparing coda consonant mispronunciations to vowel mispronunciations. In the current study, we do not examine infants’ preferences for consonants or vowels, but instead examine the neural bases of consonant and vowel processing by presenting infants with onset consonant (e.g., cave /kav/ - gave /gav/), coda consonant (e.g., taube /tob/ - / taupe /top/) and vowel (e.g., gate /gat/ - /gette /gεt/) mispronunciations of newly segmented words and measuring ERP responses to their presentation. By comparing the ERP responses to mispronounced words to that of target or control words, we can examine whether infants process these mispronunciations similarly to target (not sensitive to the mispronunciation) or control (sensitive to the mispronunciation) words. Based on evidence from behavioral studies with infants [13], we may expect infants to be sensitive to onset and coda consonant—but not medial vowel—mispronunciations. 

In contrast to behavioral evidence, ERP evidence may provide a finer examination of sensitivity to consonant and vowel mispronunciations. Previous studies with adults have found sensitivity to both consonant and vowel mispronunciations in auditory processing—both with and without noise [30,31]—as well as in visual word recognition, but at different timing and scalp distributions [32,33,34]. ERP studies with infants have not directly compared consonant and vowel processing, but a series of auditory word recognition studies have examined consonant or vowel processing in older infants. Separate studies reveal that English-learning 14-month-olds show sensitivity to vowel [35,36] but not consonant mispronunciations of familiar words, although this sensitivity has developed by 20 months [37]. In regards to vowel mispronunciation sensitivity, Duta et al. [36] found an early, *P2*-like response (225–325 ms), whereas Mani et al. [35] found a N400-like response (200–300 ms, 400–600 ms). In contrast, the sensitivity to consonant mispronunciations found in 20-month-olds was reflected by both P100- and the N200–N400-like components. Across these studies, components began as early as 200 ms, but differed in their relative polarity (positive or negative) and scalp distribution. Furthermore, this set of studies examined the phonological detail of stored familiar word representations, and in the case of the vowel mispronunciation studies [35,36], also presented picture references. 

In the current study, we examine for the first time ERP responses to mispronunciations of newly segmented words without any visual references. Mispronunciation processing in this case reflects a comparison between the mispronunciation and the newly segmented target word. Based on previous segmentation studies with ERPs, mispronunciation sensitivity will be reflected by a difference between target and mispronounced words. In regards to consonants and vowels, we may find sensitivity to consonant, but not vowel, mispronunciations based on previous behavioral evidence. By measuring ERPs, however, we may also find differences in the timing of response, due to the relative position of the mispronounced phonemes (onset, medial, or coda position) as well as distribution of effects, where more localized, or left-lateralized, effects may reflect developmental maturity [37]. Furthermore, although the C-bias has been established using onset or coda consonant mispronunciations with eight-month-old infants in word segmentation [13], ERPs might allow us to capture small temporal differences in the response to mispronunciations at the onset and coda of the word.

Previous studies investigating the emergence of the C-bias have examined group means to establish that it is learned between six and eight months (in French and possibly also Italian). Within these groups, however, some infants may have developed a C-bias by six months while others may still have a V-bias at eight months. Hence, we may expect individual variation in the relative sensitivity to consonant and vowel mispronunciations within a group of infants of the same age. This may be reflected by differences between Negative and Positive Responders in their processing of consonant and vowel mispronunciations, which would suggest that maturity in segmentation skill is related to mispronunciation sensitivity. Furthermore, since it has been proposed that the C-bias bootstraps language acquisition [16], infants who discover the importance of consonants in lexical processing at an earlier age may benefit from this information, which may be evident in more advanced lexical skills than their peers who developed this bias at a later age. While links have been found between individual variation in early phonetic processing performance and later language outcomes [19], we investigate here whether sensitivity to consonant and vowel mispronunciation is related to vocabulary growth in the second year of life, and whether this effect is stronger for consonant mispronunciations, which would support the link between the C-bias and lexical development.

## 2. Materials and Methods

### 2.1. Participants

Thirty-two eight-month-old infants were included in the final sample (mean age = 258.18 days, age range = 244–274 days, 14 females). All participants were healthy, full term, French-learning monolinguals recruited from the Paris metropolitan area through birth lists. Parents gave written informed consent for inclusion before they participated in the study. The study was conducted in accordance with the Declaration of Helsinki, and the Ethics Committee of CERES (N°2011-14, approved 18 October 2011) approved the protocol. Eight additional infants were tested but not included in the final sample due to providing too few artifact-free trials.

### 2.2. Stimuli

A female native speaker of French recorded the stimuli in a sound-attenuating booth, using a mild infant-directed register. The stimuli consisted of 252 monosyllabic French CVC words produced in isolation as well as embedded within sentences. These words were chosen because of their low frequency, based on frequency analyses (*M* = 32.93 tokens per million) in the adult-directed French corpus Lexique [38]. Although 14 of the words (e.g., beurre, poule, tasse) are listed in the French version of the MacArthur-Bates Communicative Development Inventory (MCDI) [39], an analysis of responses from parents of eight-month-olds collected previously in our laboratory (*n* = 41) estimates that these words are known by less than 50% of infants at this age (*M* = 5.29%; range 0–39%). Each word served as a target, mispronunciation, and control word across conditions, therefore limiting any individual effects one item may have on the results. For each isolated word and sentence, one token was selected. All words had a minimal pair present in the stimuli set, differing by one feature in the onset or coda consonant (voicing, place, or manner of articulation) or in the medial vowel (height, roundness, or place). Across infants, and to control for potential word frequency effects, each word served once as a target word, once as a one-feature mispronunciation, and once as a phonologically unrelated control word. 

Eight sentences were created as frames around each of the 252 words, in which the target word appeared once (leading to a total of 2016 sentences. Each sentence had on average 8.52 syllables (standard deviation (*SD*) = 0.67; range 8–11). To avoid any cues given by pauses between sentences, target words never appeared in the absolute first or last positions. The onset of each phoneme (onset consonant, medial vowel, and coda consonant) for each target word in each sentence and for all words in the Test Phase was determined using the EasyAlign plugin [40] for Praat [41] and verified by hand. This timing information was used during the experiment to send event codes time-locked to word-onset and individual phoneme-onset in both isolated and sentence embedded words. Words were on average 565 ms (range 370 to 770). Regarding individual phoneme duration within words, on average the onset consonant lasted 129 ms (range 34 to 216), the medial vowel lasted 244 ms (range 106 to 362), and the coda consonant lasted 202 ms (range 91 to 473). Within the word, the onset for phonemes was on average 129 ms (range 34 to 216) for medial vowels and 373 ms (range 180 to 509) for coda consonants. Table 1 gives examples of sentences from the Familiarization Phase and examples of target, mispronunciation, and control words from the Test Phase.

### 2.3. Procedure

We used a modified version of the procedure used by Männel and Friederici [2]. The experiment consisted of 63 Familiarization—Test blocks. First, infants heard eight different sentences containing the same target word (Familiarization Phase), followed by nine randomly presented isolated words (Test Phase). The isolated words were three repetitions each (same token) of the target word, a one-feature mispronunciation, and a control word. Interstimuli intervals in the Test Phase were jittered (range 300–600 ms).

All infants heard three different kinds (21 each) of Familiarization—Test blocks, differing on the type of mispronunciation present in the Test Phase: onset consonant, medial vowel, or coda consonant. Consonant mispronunciations differed by voicing, place, or manner of articulation (7 each) and vowel mispronunciations differed by height, roundness, or place (7 each). Order of blocks was randomized. Four different counterbalancing versions were created such that across versions (which were presented to different infants), each word served once as a target, once as a mispronunciation, and once as a control word. Infants heard each word in only one Familiarization-Test block; words were never repeated within a version. An example test set for one counterbalancing version can be found in the Supplementary Materials (Table S1).

The experiment took place in a sound-attenuating booth and lasted about 27 min. The infants sat on one of their parents’ lap. Two loudspeakers, situated approximately 1 meter in front of the infant, presented the stimuli at a comfortable listening level. Stimuli were presented using the E-Prime 2.0 software (Psychology Software Tools, Pittsburgh, PA, USA) [42]. In order to keep the infants interested and still, they were presented with small, silent, plastic toys or peek-a-boo. No masking method was used for the parents during the experiment in order to not further increase infants’ stress level. However, parents were asked not to talk during the experimental study. Breaks were taken when necessary and programmed to only occur after the completion of a Familiarization—Test block. 

### 2.4. Vocabulary Questionnaire

At the time of their visit, parents were asked to complete the French Communicative Developmental Inventory Words and Gestures for ages eight to 16 months [39]. To examine how infants’ vocabulary scores grew with time, parents were also asked to complete the same questionnaire when their child was 13 months, as well as the French CDI (Communicative Development Inventory) Words and Phrases for ages 16 to 30 months when their child was 16 and 24 months. Of the 32 infants that participated in the ERP experiment and were included in the final sample, 26 returned completed vocabulary questionnaires at the time of test (mean words produced = 0.85; *SD* = 1.64; range = 0–7). Vocabulary questionnaires were returned by 26 infants at 13 months (mean words produced = 2.70; *SD* = 3.18; range = 0–11), 26 infants at 16 months (mean words produced = 15.20; *SD* = 15.25; range = 0–55), and 22 infants at 24 months (mean words produced = 229.50; *SD* = 139.00; range = 43–531). There were 14 infants who completed vocabulary questionnaires at all 4 ages, 25 infants who completed vocabulary questionnaires at 3 ages, 27 infants who completed vocabulary questionnaires at 2 ages, and 31 infants who completed vocabulary questionnaires at least at one age. For each infant, we calculated total number of words produced at each age measured. We examined words produced, in contrast to other vocabulary measurements, as this is the only category measured on both versions of the French CDI.

### 2.5. EEG Recording and Preprocessing

Electro-encephalography was recorded from a 128 channel HydroCel Geodesic Sensor Net (EGI) [43] using an EGI NetAmps 400 amplifier (Electrical Geodesics Inc., Eugene, OR, USA). Electrodes were placed according to the EGIS system. Electrodes were referenced online to the vertex. EEG signals were recorded using a sampling rate of 250 Hz. Impedance of all electrodes was kept below 50 kΩ. Participant files were exported using the Netstation Software (Electrical Geodesics Inc., Eugene, OR, USA) to be further analyzed using custom-made Matlab scripts using functions from the EEGLAB [44] and ERPLAB [45] toolboxes.

The EEG signal was first checked for bridging, using the EBridge plugin [46] for the EEGLAB toolbox. One participant in the final dataset had 5 bridged channels, which were interpolated in a later step. The signal was then filtered using a 0.3 to 30 Hz bandpass filter. Bad channels were examined visually, as well as using a kurtosis threshold of −5 to 5. Channels on the outer electrode rings that were marked as bad channels in 12 or more subjects were marked for removal, as well as their hemispheric pairs. This included 33 electrodes. These 33 channels were removed from the unreferenced EEG signal and the bad channels identified in a previous step were replaced using spherical–spline interpolation. No participant had more than 12 interpolated channels. The EEG signal was re-referenced to the average reference—which is considered the best reference choice for high density recordings as it gives an estimate of noise that is not biased to a specific location [47]—and segmented to create epochs from 200 ms before and 1000 ms after the onset of the word (or phoneme, in the phoneme–onset analyses). A simple voltage threshold of −200 to 200 μV was used to reject trials containing artifacts. For each infant, we calculated averaged, baseline corrected waveforms for each condition. To contribute to the analysis, a participant had to provide at least 10 trials per condition. For the Familiarization Phase, infants contributed an average of 53.03 trials (range: 29–99; maximum possible 126) for the first two (initial) sentence tokens and an average of 50.03 trials (range: 21–93; maximum 126) for the last two (final) sentence tokens. For the Test Phase, infants contributed an average of 78.09 trials (range: 39–157; maximum 189) for target words and 76.94 trials (range: 38–159; maximum 189) for control words. On average, infants contributed 25.72 trials (range: 12–50; maximum 63) for onset consonant mispronunciations, 25.31 trials (range: 14–46; maximum 63) for medial vowel mispronunciations, and 26.81 trials (range: 12–48; maximum 63) for coda consonant mispronunciations.

### 2.6. ERP Data Analysis

For both the Familiarization and Test Phases, we created averaged ERPs time-locked to word onset. We used ggplot2 [48] to plot averaged ERP waveforms and *erpR* [49] to plot topography maps of time windows of interest. For the Familiarization Phase, we compared the first two sentence tokens (1 and 2; initial) with the last two sentence tokens (7 and 8; final). This allowed for an examination of memory trace formation to the segmented target word [2,18]. For the Test Phase, we compared the responses of target and control words to examine the word familiarity effect. To examine sensitivity to mispronunciations and capture potential temporal and scalp distribution differences related to word position, we compared the ERP response to target and mispronounced words for each kind of mispronunciation separately (onset consonant, medial vowel, coda consonant). Similar to previous studies [4,10,11,18], the onset of an effect was determined by comparing the difference wave between the two conditions of comparison (e.g., initial–final words; target–control words; target–mispronounced words) with chance (=0). For each electrode, a two-tailed *t*-test was calculated to determine where conditions differed significantly on at least five consecutive bins of 50 ms with a 40ms overlap (i.e., 0–50, 10–60 etc.). The offset of the effect was determined at the point where this difference on consecutive bins was no longer significant.

**Figure 1:**
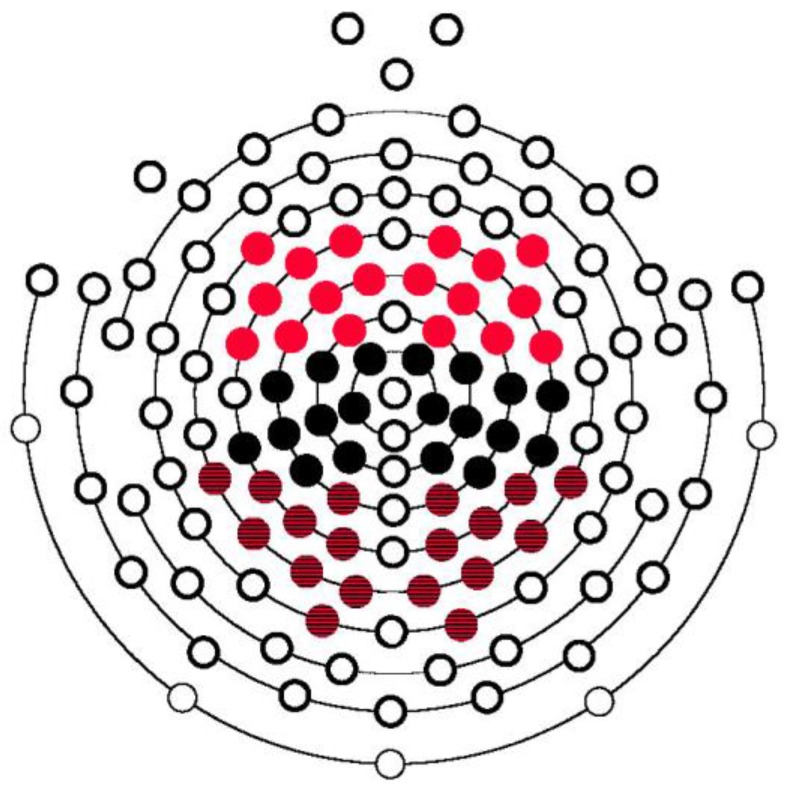
Position of electrodes included in final event-related potentials (ERP) data analysis. Electrodes highlighted in red represent left and right anterior electrodes, in black left and right central electrodes, and in striped red and black left and right posterior electrodes.

For the purposes of data reduction, a selection of 54 electrodes was entered into data analysis. Similar to Männel and Friederici [2], these electrodes were divided into six regions of interest (ROIs) with 9 electrodes each: left anterior, right anterior, left central, right central, left posterior, and right posterior. Three separate sets of repeated measures ANOVAs were conducted using the R package *ez* [50]. Figure 1 plots the positions of electrodes in each ROI. First, we examined the formation of a memory trace in the Familiarization Phase with the factors Word (initial, final), Hemisphere (left, right), and Region (anterior, central, posterior). Second, we examined the word familiarity effect in the Test Phase with the factors Word (target, control), Hemisphere, and Region. Finally, we separately examined sensitivity to each type of mispronunciation in the Test Phase with the factors Word (target, mispronunciation, control), Hemisphere, and Region. Our hypothesis predicted differences between onset consonant, medial vowel, and coda consonant mispronunciations, including potential timing and scalp distribution differences resulting from differences in the type (consonant, vowel) and position of the mispronunciation (onset, medial, coda). Analyses for onset consonant, medial vowel, and coda consonant mispronunciations were conducted separately in order to capture these potential differences. Furthermore, to ensure that effects were due to mispronunciations and not differences in phoneme onsets across items, we conducted phoneme-locked analyses for both medial vowel and coda consonant mispronunciations. If a main effect of or interaction with Word was found, follow-up ANOVAs and post-hoc *t*-tests were conducted to determine the pattern of effects.

To examine links between response polarity in the Test Phase and responses in both the Familiarization Phase and to mispronunciations, we used the mean difference over left anterior electrodes between target and control words in the Test Phase to categorize infants as Negative or Positive Responders, similar to previous studies [10,18]. ANOVA analyses with Responder (Negative, Positive) as a between-subjects factor were conducted, as well as follow-up ANOVA and *t*-tests to determine the pattern of effects.

To examine how ERP responses at test were related to vocabulary growth, we used growth curve analysis [23,24] to analyze the relationship between the growth of word production at 8, 13, 16, and 24 months and effects of both word familiarity and mispronunciation sensitivity. We started with a null model that included the dependent variable of productive vocabulary (*z*-score transformed) and participants as random factors; we added the predictor variable or variables to this model (incrementally, if there was more than one predictor) to see whether the model was improved. Model fit was assessed using chi-square tests on the log-likelihood values to compare different models.

For the effect of word familiarity, the ages at which word production was measured was modeled with a third-order (cubic) orthogonal polynomial and fixed effects of Response Polarity (Negative, Positive) on all time terms. Positive Responders were coded as the reference condition. For the effect of mispronunciation sensitivity, we used the ERP response for each type of mispronunciation (early time window) to calculate a mispronunciation sensitivity difference score by subtracting mispronunciation from target responses. We focused on ERP responses in the left hemisphere, as this was the only region that showed an effect of mispronunciation sensitivity in the early time window (Negative Responders, onset consonant mispronunciations, see Results below). The ages at which word production was measured was modeled with a third-order (cubic) orthogonal polynomial and fixed effects of mispronunciation sensitivity and mispronunciation type (onset consonant, medial vowel, coda consonant) on all time terms. Both models also included participant random effects on all time terms except the cubic (estimating random effects is “expensive” in terms of the number of observation required, so this cubic term was excluded because it tends to capture less-relevant effects in the tails). *Z*-scores of total number of words produced were calculated on the entire sample and served as the dependent variable for both analyses. 

## 3. Results

### 3.1. Familiarization Phase: Memory Trace Formation

**Figure 2:**
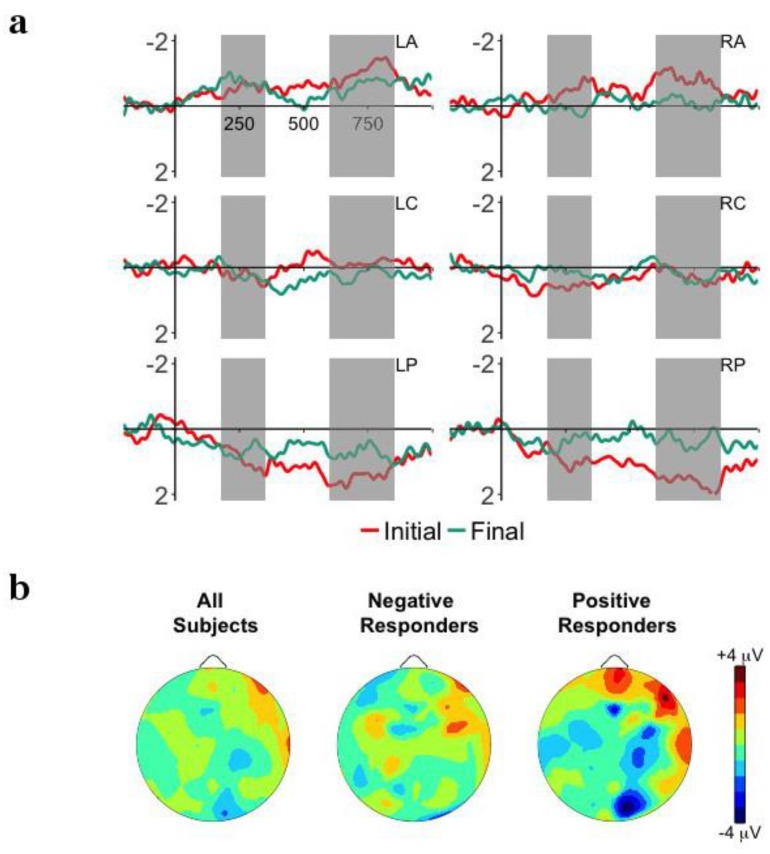
(**a**) Grand-average event-related potentials (ERP) responses time-locked to word onset for the first two (1st/2nd) and last two (7th/8th) tokens of the embedded target words in the Familiarization Phase for the 6 ROIs: left anterior (LA), right anterior (RA), left central (LC), right central (LR), left posterior (LP), right posterior (RP). The time windows of interest, 180–350 ms and 600–850 ms are highlighted in gray. (**b**) Mean distribution plots for the ERP effect of memory trace formation in the Familiarization Phase (7th/8th tokens—1st/2nd) in the 180–350 ms time window for overall group performance (left), Negative Responders (central), and Positive Responders (right).

Figure 2a plots the ERP waveforms for the first two (initial) and last two (final) instances of the target word embedded within the sentences presented in the Familiarization Phase. Onset analyses revealed an early negative effect over left anterior and posterior electrodes from 180 to 350 ms, and a late positive effect for right anterior electrodes from 600 to 850. However, there was no main effect of or interaction with Word in either the early (*p*’s > 0.12) or late (*p*’s > 0.1) time windows. This fails to provide evidence that infants have established a memory trace for the target word (but see following analysis). 

### 3.2. Test Phase: Word Familiarity Effect

**Figure 3:**
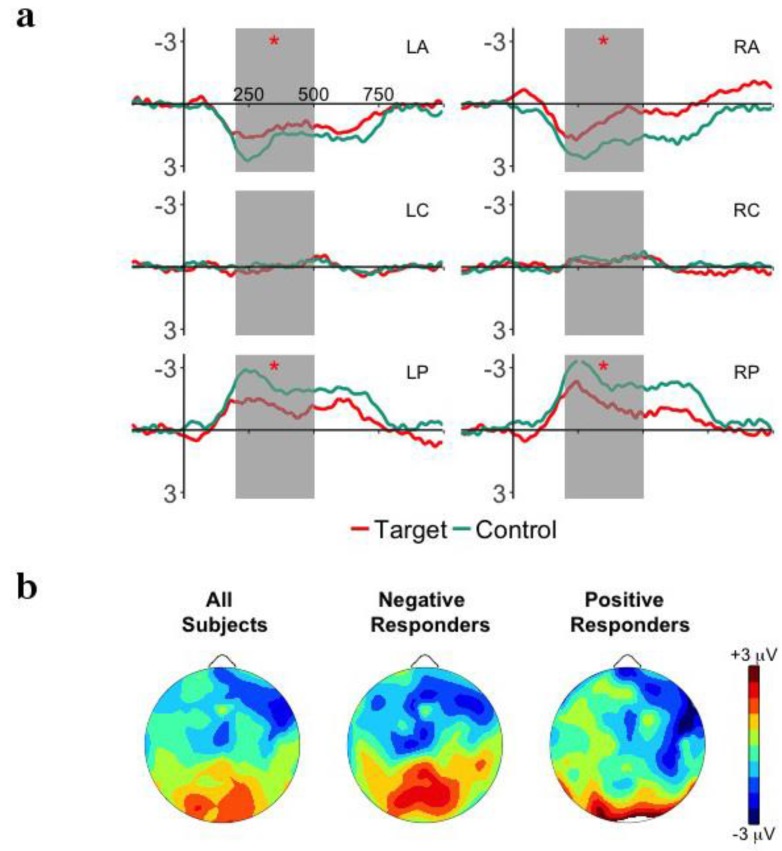
(**a**) Grand-average event-related potentials (ERP) responses time-locked to word onset for the target and control words presented in the Test Phase for the 6 ROIs: left anterior (LA), right anterior (RA), left central (LC), right central (LR), left posterior (LP), right posterior (RP). The time window of interest, 200–500 ms is highlighted in gray and significant effects are marked as * *p* < 0.05. (**b**) Mean distribution plots for the ERP effect of word familiarity in the Test Phase (target—control words) in the 200–500 ms time window for overall group performance (left), Negative Responders (central), and Positive Responders (right).

Figure 3a plots the ERP waveforms for target and control words presented in the Test Phase. Onset analyses revealed a negative deflection over left anterior electrodes from 200 to 350, a negative effect over right anterior electrodes from 250 to 500 or 800 ms, and a positive effect over posterior electrodes from 200 to 600 ms. We chose to examine the time window of 200–500 ms because this covered the most electrodes and is similar to time windows previously used (e.g., [11]). The main effect of Word was not significant (*p* > 0.24), but the Word X Region interaction was significant, *F*(2,62) = 4.63, *p* = 0.031, η^2^*_p_* = 0.13. All other interactions with Word were not significant (*p*’s > 0.5). Post-hoc analyses revealed that target words were significantly more negative than control words over anterior electrodes, *t*(31) = −2.57, *p* = 0.015, *d* = −0.64, and significantly more positive over posterior electrodes, *t*(31) = 2.12, *p* = 0.042, *d* = 0.53. There was no difference over central electrodes (*p* = 0.67). This word familiarity effect suggests that infants recognized the target words presented in the Test Phase, and implies that they had segmented them from the passages in the Familiarization Phase.

### 3.3. Relationship between Familiarization and Test Phases

Infants showed no apparent evidence of segmentation in the Familiarization Phase, although they did show a word familiarity effect with negative polarity over anterior electrodes in the Test Phase. Following the results of Junge et al. [18], we may expect that performance in the Familiarization and Test Phases may be related. We first classified infants by the polarity of their response in the Test Phase (target-control words) over left anterior electrodes. Figure 3 plots the distribution of mean ERP responses in the Familiarization (2b) and Test Phases (3b) for Negative and Positive Responders. A reanalysis of the Test Phase shows, for Negative Responders (*n* = 19; *M* = −2.20 μV; *SD* = 1.46), a significant Word X Hemisphere interaction, *F*(1,18) = 5.96, *p* = 0.025, η^2^*_p_* = 0.25, as well as Word X Region, *F*(2,36) = 12.55, *p* = 0.001, η^2^*_p_* = 0.41. For Positive Responders (*n* = 13; *M* = +1.63; *SD* = 0.75), the Word X Hemisphere interaction was significant, *F*(1,12) = 6.01, *p* = 0.031, np2 = 0.33. 

We then examined the relationship between polarity response in the Test Phase and performance in the Familiarization Phase, the early and late time windows investigated in the Familiarization Phase were once again subjected to a repeated-measures ANOVA, including the between-subjects factor of Responder (negative, positive). In the early time window, the Responder X Word X Hemisphere interaction was significant, *F*(1,30) = 4.58, *p* = 0.041, η^2^*_p_* = 0.13. Separate analysis show that for Negative Responders, the Word X Hemisphere interaction approached significance, *F*(1,18) = 4.00, *p* = 0.061, η^2^*_p_* = 0.18, with a more negative response over left hemisphere electrodes to the last two tokens compared to the first two. For Positive Responders, neither the main effect nor interactions were significant (*p*’s > 0.25). In the later time window, there were no significant main effect of or interaction with Word. Hence, infants who showed a more mature response in the Test Phase (Negative Responders) also showed some evidence of segmentation in the Familiarization Phase.

### 3.4. Relationship between Word Familiarity Effect and Vocabulary Growth

**Figure 4:**
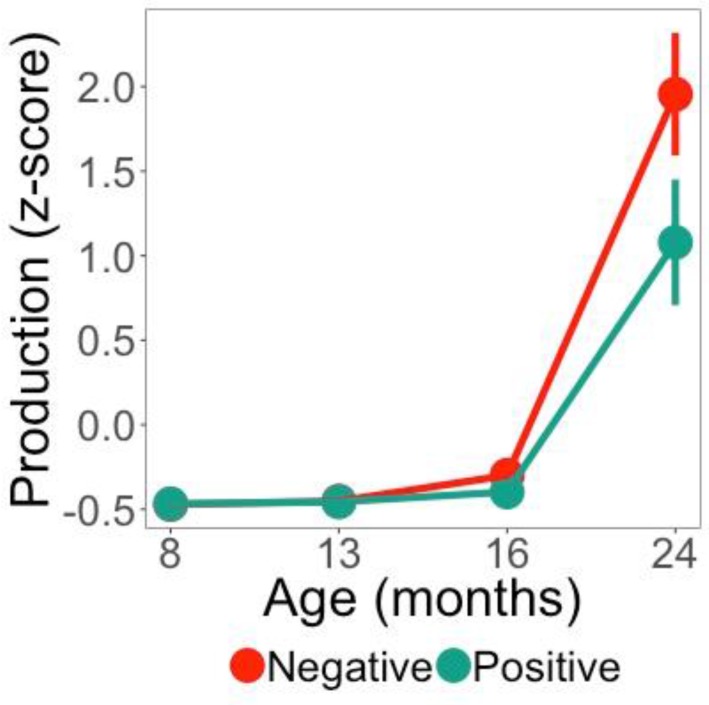
Total word production (*z*-score) at the four ages measures (8, 13, 16, 24 months) for both Negative and Positive Responders. Lines indicate the fit of the model and whiskers indicate a standard error of 1.

We next examined whether Negative and Positive Responders showed differences in productive vocabulary growth. The best fitting model included an interaction between the three time terms (linear, quadratic, and cubic) and Response Polarity (Negative or Positive). Removing Response Polarity from the model significantly decreased the goodness of fit, as indicated by likelihood ratio tests—effect of Response Polarity: χ^2^(4) = 9.99, *p* = 0.04. The output for the growth curve model is given in the Supplementary Materials (Table S2). Figure 4a depicts production growth with model fits for the effect of Response Polarity at each age measured. There was a main effect of Response Polarity, indicating that Negative Responders had a significantly greater productive vocabulary than Positive Responders (*Estimate* = −0.25, *SE* = 0.117, *p* < 0.05). A significant interaction between Response Polarity and the linear time term indicated steeper vocabulary growth for Negative compared to Positive Responders (*Estimate* = −0.62, *SE* = 0.302, *p* < 0.05). A significant interaction between Response Polarity and the cubic time term indicated that the asymmetric growth (reflected in the rather flat curve at earlier ages and rapid growth at 24 months) was greater for Negative than Positive Responders (*Estimate* = −0.142, *SE* = 0.055, *p* < 0.01). Infants who showed a more mature response in the Test Phase (Negative Responders) had greater productive vocabulary growth from eight to 24 months.

### 3.5. Test Phase: Sensitivity to Mispronunciations

**Figure 5:**
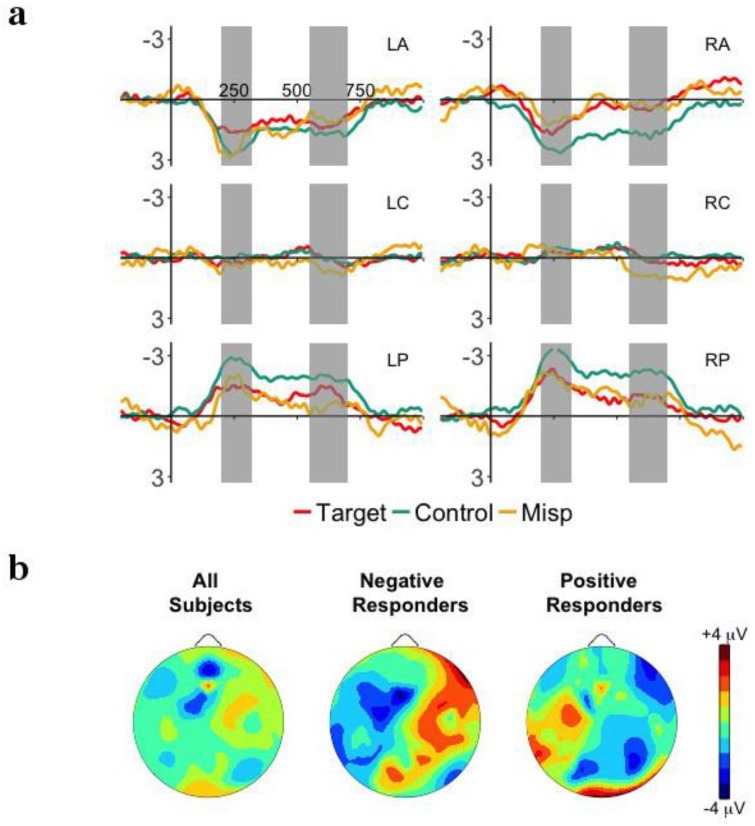
(**a**) Grand-average event-related potentials (ERP) responses time-locked to word onset for target words and onset consonant mispronunciations in the Test Phase for the 6 ROIs: left anterior (LA), right anterior (RA), left central (LC), right central (LR), left posterior (LP), right posterior (RP). The time windows of interest, 200–320 ms and 550 to 700 ms are highlighted in gray. (**b**) Mean distribution plots for the ERP effect of mispronunciation sensitivity in the Test Phase (target words—onset consonant mispronunciations) in the 200–320 ms time window for overall group performance (left), Negative Responders (central), and Positive Responders (right).

**Figure 6:**
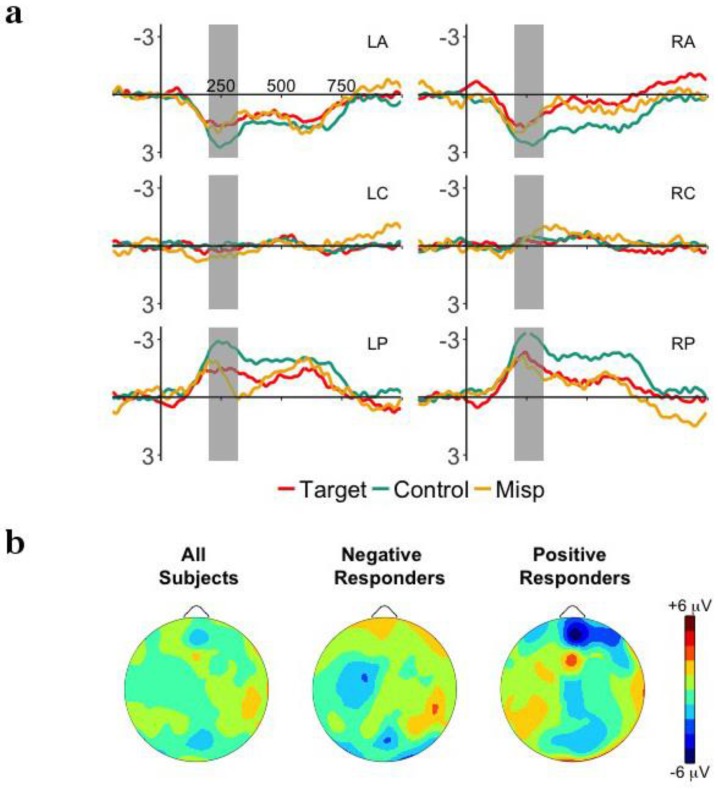
(**a**) Grand-average event-related potentials (ERP) responses time-locked to word onset for target words and medial vowel mispronunciations in the Test Phase for the 6 ROIs: left anterior (LA), right anterior (RA), left central (LC), right central (LR), left posterior (LP), right posterior (RP). The time window of interest, 200–320 ms is highlighted in gray. (**b**) Mean distribution plots for the ERP effect of mispronunciation sensitivity in the Test Phase (target words—medial vowel mispronunciations) in the 200–320 ms time window for overall group performance (left), Negative Responders (central), and Positive Responders (right).

**Figure 7:**
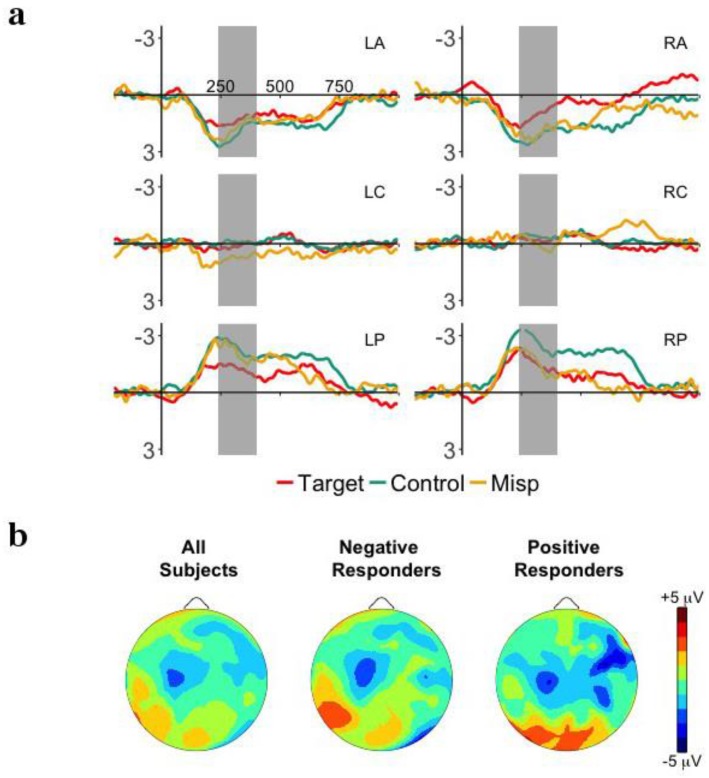
(**a**) Grand-average event-related potentials ERP responses time-locked to word onset for target words and coda consonant mispronunciations in the Test Phase for the 6 ROIs: left anterior (LA), right anterior (RA), left central (LC), right central (LR), left posterior (LP), right posterior (RP). The time window of interest, 200–400 ms is highlighted in gray. (**b**) Mean distribution plots for the ERP effect of mispronunciation sensitivity in the Test Phase (target words—coda consonant mispronunciations) in the 200–320 ms time window for overall group performance (left), Negative Responders (central), and Positive Responders (right).

Figure 5, Figure 6 and Figure 7 plot the ERP waveforms for target, mispronounced, and control words, for each type of mispronunciation (onset consonant, medial vowel, coda consonant). Visual inspection identifies effects over different clusters of electrodes and time windows for each mispronunciation type, roughly showing more negative responses over anterior electrodes from about 200 to 400 ms. In the following sections, we consider each mispronunciation type separately, in order to identify potential differences in the responses.

#### 3.5.1. Onset Consonant Mispronunciations

Figure 5a plots the ERP waveforms for targets, onset consonant mispronunciations, and control words presented in the Test Phase. Onset analysis comparing targets and mispronunciations revealed a negative deflection over left anterior electrodes from about 200 to 320 ms and 550 to 700 ms, and a longer, negative effect from 350 to 700 ms for left and right posterior electrodes. We chose the time windows 200–320 ms and 550–700 ms to further investigate these effects. For the early time window, the interaction between Word and Region approached significance, *F*(4,124) = 2.59, *p* = 0.075, η^2^*_p_* = 0.08. The main effect of Word (*p*’s > 0.11) and all other interactions with Word were not significant (*p*’s > 0.23) for either the early or late time windows. Analyzed as a group, no sensitivity to onset consonant mispronunciations could be revealed.

To examine whether detection of onset consonant mispronunciations is modulated by word familiarity performance, the previous ANOVA was repeated adding Responder as a between-subjects factor. Figure 5b plots the ERP responses to target, onset consonant mispronunciations and control words for Negative and Positive Responders. In the earlier time window (200–320 ms), there was a significant interaction between Responder, Word, and Hemisphere, *F*(2,60) = 7.50, *p* = 0.003, η^2^*_p_* = 0.20, as well as between Responder, Word, and Region, *F*(4,120) = 3.49, *p* = 0.027, η^2^*_p_* = 0.10. For Negative Responders, follow-up ANOVAs revealed a significant interaction between Word and Hemisphere, *F*(2,36) = 4.84, *p* = 0.030, η^2^*_p_* = 0.21, as well as Word and Region, *F*(4,72) = 6.07, *p* = 0.004, η^2^*_p_* = 0.25. Post-hoc analyses revealed that Negative Responders had significantly more negative responses to targets compared to onset consonant mispronunciations on left, *t*(18) = −2.76, *p* = 0.013, *d* = −0.89, but not right hemisphere electrodes (*p* > 0.07). There was no difference between onset consonant mispronunciations and control words on electrodes from either hemisphere (*p*’s > 0.07). The follow-up analysis with Positive Responders revealed no significant effect of or interaction with Word (*p*’s > 0.06). In the later time window (550–700 ms), there was a significant interaction between Responder, Word, and Hemisphere, *F*(2,60) = 8.29, *p* = 0.002, η^2^*_p_* = 0.22. For Negative Responders, there was a significant interaction between Word and Hemisphere, *F*(2,36) = 3.67, *p* = 0.05, η^2^*_p_* = 0.17. Post-hoc analyses revealed that Negative Responders had significantly more negative responses to targets compared to onset consonant mispronunciations on left, *t*(18) = −2.56, *p* = .020, *d* = −0.83, but not right hemisphere electrodes (*p* > 0.12). Subsequent post-hoc analyses revealed no significant differences between onset consonant mispronunciations and control words (*p*’s > 0.11). For Positive Responders, there was a significant interaction between Word and Hemisphere, *F*(2,24) = 4.42, *p* = 0.023, np2 = 0.27. Responders had significantly more positive responses to targets compared to onset consonant mispronunciations on left hemisphere electrodes, *t*(12) = 2.83, *p* = 0.015, *d* = 1.11, while responses were more negative to targets compared to onset consonant mispronunciations on right hemisphere electrodes, *t*(12) = −2.19, *p* = 0.049, *d* = −0.86. Subsequent post-hoc analyses revealed no significant differences between onset consonant mispronunciations and control words (*p*’s > 0.19).

When analyzed as a group, infants did not show sensitivity to onset consonant mispronunciations. When word familiarity performance (Negative vs. Positive responders) was taken into account, however, sensitivity was revealed. Specifically, effects for Negative Responders were found in both the early and late time windows, with a more negative response to targets compared to onset consonant mispronunciations over left hemisphere electrodes. Effects for Positive Responders were restricted to the late time window, with a negative response to target compared to onset consonant mispronunciations over left hemisphere electrodes and a positive response to target compared to onset consonant mispronunciations over right hemisphere electrodes.

#### 3.5.2. Medial Vowel Mispronunciations

Figure 6a plots the ERP waveforms for targets, medial vowel mispronunciations, and control words presented in the Test Phase. Onset analysis comparing targets and mispronunciations showed that the early effect was finished by 320 ms. We chose the time window 200–320 ms to further investigate these effects. Both the main effect of Word, *F*(2,62) = 3.06, *p* = 0.07, η^2^*_p_* = 0.09, and the interaction between Word and Region, *F*(4,124) = 2.72, *p* = 0.06, η^2^*_p_* = 0.08, approached significance. All other interactions with Word were not significant (*p*’s > 0.79). Analyzed as a group, no sensitivity to medial vowel mispronunciations could be revealed.

To examine whether detection of medial vowel mispronunciations is modulated by word familiarity performance, the previous ANOVA was repeated adding Responder as a between-subjects factor. Figure 6b plots the ERP responses to targets, medial vowel mispronunciations, and control words for Negative and Positive Responders. There was a significant interaction between Responder, Word, Hemisphere, and Region, *F*(4,120) = 3.11, *p* = 0.035, η^2^*_p_* = 0.09. Follow-up ANOVAs showed that the interaction between Word and Region was significant for Negative Responders, *F*(4,72) = 5.39, *p* = 0.006, η^2^*_p_* = 0.23. Subsequent post-hoc analyses revealed that medial vowel mispronunciations were significantly more negative than control words over anterior electrodes, *t*(18) = 2.38, *p* = 0.028, *d* = 0.77, and significantly more positive than control words over posterior electrodes, *t*(18) = −3.78, *p* = 0.001, *d* = −1.23; medial vowel mispronunciations did not differ from target words in any region. The follow-up analysis with Positive Responders revealed no significant effect of or interaction with Word (*p*’s > 0.17).

When analyzed as a group or when phoneme position (word vs. mispronounced phoneme onset) was taken into account, infants did not show sensitivity to medial vowel mispronunciations. When word familiarity performance (Negative vs. Positive responders) was taken into account, however, Negative Responders were found to respond differently to medial vowel mispronunciations and control words.

#### 3.5.3. Coda Consonant Mispronunciations

Figure 7a plots the ERP waveforms for targets, coda consonant mispronunciations, and control words presented in the Test Phase. Onset analysis comparing targets and mispronunciations showed that this effect began at 240 ms and was finished by 400 ms. We chose the time window 240–400 ms to further investigate these effects. The main effect of Word approached significance, *F*(2,62) = 2.98, *p* = 0.077, η^2^*_p_* = 0.09. All other interactions with Word were not significant (*p*’s > 0.30). Analyzed as a group, no sensitivity to coda consonant mispronunciations could be revealed.

To examine whether detection of coda consonant mispronunciations is modulated by word familiarity performance, the previous ANOVA was repeated adding Responder as a between-subjects factor. Figure 7b plots the ERP responses to targets, coda consonant mispronunciations and control words for Negative and Positive Responders. The interaction between Responders, Word, and Hemisphere approached significance, *F*(2,60) = 2.69, *p* = 0.094, η^2^*_p_* = 0.08, but there were no significant interactions between Word and Responder and any other factors (*p*’s > 0.1). 

To ensure that this pattern of results was not due to time-locking ERPs to the word onset, we conducted the same set of analyses with ERP responses time-locked to the onset of the coda consonant in target, coda consonant mispronunciations, and control words. The main effect of Word and all interactions with Word were not significant (*p*’s > 0.47). There were no significant interactions between Word and Responder and any other factors (*p*’s > 0.17).

When analyzed as a group, infants did not show sensitivity to coda consonant mispronunciations. This pattern of results remained the same with word familiarity performance (Negative vs. Positive Responders) as well as with phoneme position (word vs. mispronounced phoneme onset).

### 3.6. Relationship between Mispronunciation Sensitivity and Vocabulary Growth

**Figure 8:**
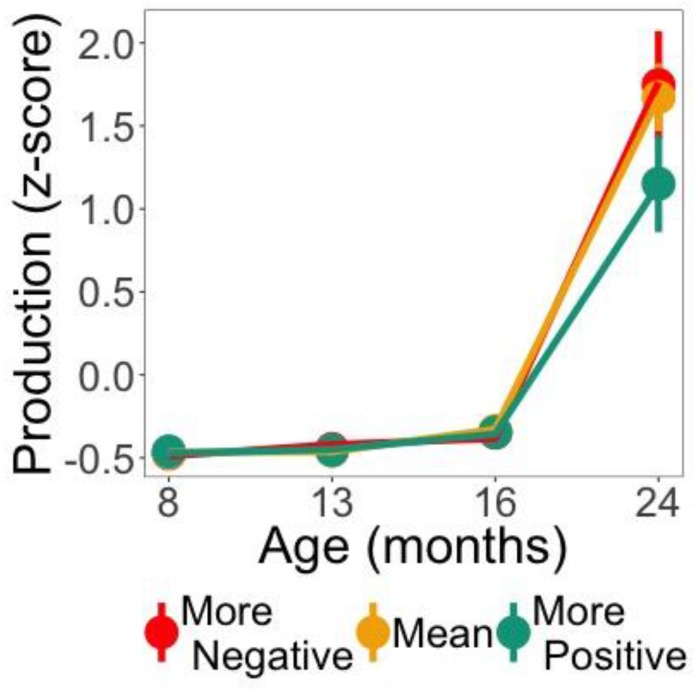
Total word production (*z*-score) at the four ages measures (8, 13, 16, 24 months) as a function of the mispronunciation sensitivity difference score (target—mispronunciation) at eight months. Participants were grouped into whether this difference score was greater than 1 SD below the group mean (More Negative), within 1 SD of the mean (Mean), or greater than 1 SD above (More Positive). Note that these data groupings by mispronunciation difference score is merely for illustrative purposes, the predictor in the model was continuous. Lines indicate the fit of the model and whiskers indicate a standard error of 1.

We next examined whether infants’ mispronunciation sensitivity predicted productive vocabulary growth. We focused on early ERP responses in the left hemisphere, as this was the only region that showed any effect of mispronunciation sensitivity in the current experiment (Negative Responders, onset consonant mispronunciations). The dependent variable, mispronunciation sensitivity, was calculated by subtracting the ERP response for each type of mispronunciation (early time window) from target responses. The best fitting model included an interaction between the three time terms (linear, quadratic, and cubic) and overall mispronunciation sensitivity. Removing mispronunciation sensitivity from the model significantly decreased the goodness of fit, as indicated by likelihood ratio tests—effect of mispronunciation sensitivity: χ^2^(4) = 16.42, *p* = 0.003. Interestingly, adding an interaction between mispronunciation sensitivity and mispronunciation type (onset consonant, medial vowel, coda consonant) did not improve the model: χ^2^(2) = 0.04, *p* > 0.9. The output for the growth curve model is shown in the Supplementary Materials (S3). Figure 8 depicts production growth with model fits for the effect of mispronunciation sensitivity at each age measured. There was a significant interaction between the cubic time term and mispronunciation sensitivity (*Estimate* = −0.03, *SE* = 0.01, *p* < 0.0001), capturing the asymmetric growth between early and late ages measured. Infants who showed a more negative mispronunciation response (target—mispronunciation) showed a significant improvement in vocabulary growth later in development, compared to infants who showed a more positive mispronunciation response. Although there was no evidence for sensitivity for the different mispronunciation types to predict vocabulary growth, this evidence suggests that early mispronunciation sensitivity is related to productive vocabulary growth, specifically in the later ages measured.

## 4. Discussion

The present study explored the neural bases of early word segmentation and early phonological (consonant versus vowel) processing. Regarding segmentation, our findings on infants’ performance in the Test Phase, showing a different response to target and control words, indicates familiarity with the target word through segmentation of the word form in the Familiarization Phase. This response was negative in polarity over anterior electrodes in a time window of 200–500 ms. Given that previous studies had shown that, at the group level, six- and seven-month-olds have a positive familiarity response [2,11], while nine- and 10-month-olds have a negative familiarity response [2,4,7,18], our findings place a negative familiarity group effect at the youngest age to date, eight months. 

In contrast, we found no difference in response to the initial two and final two sentence tokens in the Familiarization Phase. Previous studies familiarizing infants with passages containing target words have found that responses to embedded target words increase in negativity during the Familiarization Phase for infants as young as nine months [2,18], but there was no change in six-month-olds [2]. In both the current study and that of Männel and Friederici [2], however, infants showed an effect in the Test Phase, but not in the Familiarization Phase. Männel and Friederici [2] describe this pattern of results as evidence of phonological pattern matching between the word form segmented in the Familiarization Phase and the target word presented in the Test Phase. Our pattern of effects thus indirectly supports segmentation in the Familiarization Phase, although there was no direct evidence of memory trace formation. Additionally, it is not until nine months that infants exhibit evidence of memory trace formation during word segmentation [2] and 10 months of age that infants demonstrate evidence of rapid word recognition following a single exposure to a word [11]. Taken together with our results, this provides evidence that word segmentation skills are maturing during the first year of life. 

In addition to evidence for a word familiarity effect in the Test Phase as a group, we also found that the response polarity of this effect on anterior left electrodes was related to both memory trace formation in the Familiarization Phase as well as productive vocabulary growth. Based on previous studies [10,11,18], infants exhibiting a negative response to target compared to control words in the Test Phase (Negative Responders) are considered to have a more mature response based on the developmental trajectory of this effect. In the current study, this factor (Responder) interacted significantly in the Familiarization phase with Word (initial vs. final sentence tokens) and electrode Hemisphere. This is in line with the Junge et al. [18] findings of a link between response polarity at test and ERP responses in the Familiarization Phase. Follow-up tests examining the interaction separately for Negative and Positive Responders, however, did not reveal any significant differences between final and initial sentence tokens. There are a number of methodological differences between Junge et al. [18] and the current study which may account for this difference, such as recording system (high vs. low-density), choice of reference (average vs. linked mastoids), and number of electrodes recorded and subsequently analyzed. The studies also tested infants of differing ages (Junge et al. [18] tested 10-month-old infants while we tested eight-month-olds), which might reflect the progression of segmentation skills in this age period. This is supported by a previous study which found that nine-, but not six-month-olds show a response to target words in sentences which increases in negativity over the course of the Familiarization Phase [2]. From a developmental perspective, then, this negative-going effect evolves over the first year of life, from its absence at six months to clear presence at nine and 10 months. Our results may therefore capture an early stage in the development of segmentation skills and may reflect individual variation in a transition from positive to negative responses over anterior electrode sites in younger infants to older infants.

Using growth curve analysis [23,24], we also established a relationship between segmentation performance (as attested by Response Polarity in the Test Phase) and vocabulary growth. Word production for both Negative and Positive responders was within the normal range [39] and the growth between 16 and 24 months reflects the continuous acceleration in word production that is typical for the second year of life [51,52]. Yet, for Negative Responders, this rapid growth was greater than that of Positive Responders. Both Kooijman et al. [10] and Junge et al. [11] have also found that infants showing negative ERP responses (Negative Responders) in a segmentation task at younger ages have higher language skills later in development. Our study thus adds evidence supporting the claims that more negative responses over front anterior electrodes constitutes a more mature segmentation pattern, and that an early advantage in segmenting words from the speech stream may pay dividends of vocabulary knowledge later in development, as infants will use these abilities to more efficiently process language and learn words. This provides new evidence for a link between segmentation skills and later language skills from Dutch [10,11] and English [9] to French.

Coming now to the issue of phonological processing, we had predicted, based on the previous evidence of a C-bias in word segmentation in eight-month-olds [13], that infants in our study would show sensitivity to both onset and coda consonant mispronunciations, but not to vowel mispronunciations. Our results partly support this prediction and add further evidence to the literature of a C-bias developed by eight months of age. When analyzed as a group, infants showed no sensitivity to consonant and vowel mispronunciations of newly segmented words. When their segmentation performance (Negative vs. Positive Responders) was taken into account, however, infants showed sensitivity to onset consonant mispronunciations. In the early time window (200–320 ms), Negative Responders had significantly more negative responses to targets compared to onset consonant mispronounced words over left hemisphere electrodes, while Positive Responders showed no difference between target and onset consonant mispronounced words. In the later time window (500–700 ms), Negative Responders continued to exhibit a negative response over left hemisphere electrodes, while Positive Responders exhibited a positive response over left hemisphere electrodes and a negative response over right hemisphere electrodes. In one previous study examining onset consonant mispronunciation sensitivity, Mills and colleagues [37] found a more negative response to target compared to mispronunciations in 20-, but not 14-, month-olds. Similar to the response of Negative Responders in the current study, this effect was greater over left compared to right hemisphere electrode sites. Although there are considerable differences (age, task, native language) between the study of Mills and colleagues and our study, the pattern of results suggests that sensitivity to onset consonant mispronunciations may be localized to the left hemisphere. Positive Responders, in contrast, showed a late, positive effect over right hemisphere electrodes. We consider this pattern of results to indicate sensitivity to onset consonant mispronunciations in both Negative and Positive Responders, but that the left hemisphere negativity of Negative Responders may be indicative of a more mature response.

Based on adult and infant ERP data [32,33,34,35,36,37], we had predicted that our results could either show sensitivity to consonant but not vowel mispronunciations, or sensitivity to both but with differences in timing or scalp distributions. The time window identified by the onset analysis was similar for both onset consonant and medial vowel mispronunciations (200–320 ms). The comparison of the results for these two types of mispronunciations supports the prediction of sensitivity to consonant but not vowel mispronunciations; when analyzed separately by Responder type, infants showed sensitivity to onset consonant mispronunciations; we could not find evidence of sensitivity to vowel mispronunciations, whether analyzed as a group overall or separated by Responder type. Indeed, Negative Responders had different responses to medial vowel mispronunciations and control words, but not target words, suggesting that they processed targets and medial vowel mispronunciations similarly. Perhaps at this early stage of language acquisition, infants with a more mature segmentation response (Negative Responders) have learned that vowel information is not as informative as consonant information, as a result of lexical [28] or acoustic [29] properties, and therefore do not process vowel information in great detail. Extension of the current study to older infants will help determine if and when sensitivity to vowel mispronunciations emerges in later development. Moreover, since previous behavioral studies have shown that at five–six months, infants are more sensitive to vowel than consonant mispronunciations [14,25], even when tested with a word segmentation paradigm [13], it will be important to extend the present study to younger ages.

Unexpectedly, we could not find evidence of infants’ sensitivity to coda consonant mispronunciations. The response to coda consonant mispronunciations in the current study was different than onset consonant mispronunciations, where mispronunciations were processed differently than target words, showing sensitivity. However, this response was also different from that of medial vowel mispronunciations, where mispronunciations were processed similarly to target words by Negative Responders, showing a lack of sensitivity. This situates the response to coda consonant mispronunciations as somewhere in between the response to targets and controls: showing neither sensitivity nor necessarily lack of sensitivity.

Although behavioral data showed that infants at the same age are sensitive to both onset and coda consonant mispronunciations of segmented word forms when comparing them to vowel mispronunciations, sensitivity to onset consonants could not be attested when comparing them to target words (coda consonants were not tested in that condition; [13] suggesting that finding these sensitivity effects might be design-specific). Hence the present lack of a difference between coda consonant mispronunciations and targets might not mean that French-learning infants are insensitive to coda consonant mispronunciations, as we were also unable to establish lack of sensitivity through a difference between coda consonant mispronunciations and controls. These results nevertheless reveal that onsets are represented and/or processed with more detail than codas in the present experimental task—which might not have been the case in the behavioral task used in [13]—and could be more directly tested in future research by opposing onset and coda consonant mispronunciations. This is in line with data on Dutch-learning infants showing better processing of onset over coda consonants at 11 months [53]. These positional effects may also reflect the structure of the infant lexicon. TRACE simulations conducted on a set of words chosen to reflect the typical lexicon of 15- or 24-month-old British English-learning infants showed greater sensitivity to consonant than vowel mispronunciations in word recognition [54]. This may be the result of an early infant lexicon rich in consonant-onset words; when the position of consonants and vowels were manipulated such that the majority of words were vowel-initial, however, the simulations resulted in greater sensitivity to vowel compared to consonant mispronunciations. Yet, these positional effects might be age-, language-, and/or task-specific, as French-learning infants were found to be as sensitive to onset and coda consonant mispronunciations when learning new words at 20 months [55] or segmenting word forms at eight months [13]. These previous French findings, together with evidence of sensitivity to onset consonant mispronunciations by all infants and lack of sensitivity to medial vowel mispronunciations by Negative Responders, suggest that a strict positional interpretation of our data is unlikely. However, future studies manipulating the within-word position of consonants and vowels will be needed to better understand these developmental changes in the relative processing of onset and coda consonants. 

With respect to language acquisition, it has been proposed that the C-bias found in adults from many language backgrounds [56,57,58,59,60] constitutes a feature of the mature linguistic processing system (at least in the languages tested so far), whose emergence bootstraps language acquisition [16]. In the current study, however, we failed to find evidence that sensitivity to consonant but not vowel mispronunciations predicts vocabulary growth. Instead, sensitivity in general to phoneme (both consonant and vowel) mispronunciations was found to predict later vocabulary growth. Specifically, those infants who had a more negative response to target compared to mispronounced words had greater vocabulary growth in the later ages measured. Infants that are sensitive to the phonological form of newly segmented words at this early age may use this skill later when mapping labels to objects and recognizing familiar words, which ultimately translates to greater vocabulary growth later in life. 

## 5. Conclusions

In conclusion, during the second half of the first year of life, infants are sharpening both their word form segmentation skills as well as their phonological processing abilities. This is reflected here in the shift from a negative to a positive ERP response to newly segmented words, as well as sensitivity to onset consonant mispronunciations and lack of sensitivity to vowel mispronunciations by Negative Responders, with the response to coda consonants showing neither sensitivity nor lack of sensitivity. Furthermore, these gains in processing skills appear related to later developments, as attested here in the finding that more mature ERP responses for segmented words as well as sensitivity to their phonological form at eight months predicted greater productive vocabulary growth during the second year of life, consistent with an integrated view of language acquisition.

## Figures and Tables

**Table 1:** A table of stimuli examples given separately for each of the three types of mispronunciations (onset consonant, medial vowel, coda consonant). Although there were three types of feature changed for consonant (voicing, place, and manner) and vowel (height, place, and roundness) mispronunciations, we give one example of feature change for each mispronunciation type. For the familiarization phase, four sentences contained the target word positioned towards the beginning and four contained the target word position towards the end. English translations of the sentences are provided in parenthesis. For the test phase, the isolated words were presented three times each. Their IPA (International Phonetic Alphabet) transcriptions are given in parenthesis.

Mispronunciation Condition			
Onset consonant	Familiarization Phase		
(voicing change)	beginning	Cette cave à vins est un trésor. (This wine cellar is a treasure.) La cave sera pleine d’ici peu. (The cellar will be full soon.) Certaines caves ont été inondées. (Some cellars were flooded.) Les caves claires sont aujourd’hui très rares. (Clear cellars are very rare nowadays.)	
	end	J’ai découvert une cave ancienne. (I discovered an old cellar.) Ils sont entrés dans ces caves sombres. (They entered these dark cellars.) Ce pays possède des caves romaines. (This country has Roman cellars.) Tu connais quelques caves humides. (You know some humid cellars.)	
	Test Phase		
	Target	Mispronunciation	Novel
	cave (/kav/)	gave (/gav/)	reg (/rεg/)
Medial vowel	Familiarization Phase		
(height change)	beginning	Certaines guettes sont vulnérables au feu. (Some towers are vulnerable to fire.) La guette du château est très grande. (The tower of the castle is very big.) Les guettes de cette époque sont hautes. (The towers of this period are high.) Nos guettes résisteront à l’assaut. (Our towers will resist the onslaught.)	
	end	Les ennemis attaquent ces guettes fragiles. (The enemies attack these fragile towers.) Vos alliés consolident leur guette sud. (Your allies are consolidating their southern tower.) Il faut construire une guette solide. (You have to build a solid tower.) Les archers garderont des guettes fortes. (The archers will guard the tower well.)	
	Test Phase		
	Target	Mispronunciation	Novel
	guette (/gεt/)	gatte (/gat/)	fugue (/fyg/)
Coda consonant	Familiarization Phase		
(place change)	beginning	Quelques fers jolis sont à gagner. (Some pretty irons are up for grabs.) Le fer trois est difficile à manier. (The three iron is difficult to handle.) Les fers sont en ordre croissant. (The irons are in ascending order.) Plusieurs fers collants ont été volés. (Several sticky irons were stolen.)	
	end	J’aurais acheté ce fer neuf. (I would have bought this new iron.) Le vieil homme joue un fer en bois. (The old man plays a wooden iron.) On prend classiquement des fers gris. (We usually take gray irons.) Vous avez perdu ces fers prêtés. (You have lost those borrowed irons.)	
	Test Phase		
	Target	Mispronunciation	Novel
	fer (/fεr/)	fêle(/fεl/)	race (/ras/)

## Supplementary Materials

The following are available online at http://www.mdpi.com/2076-3425/8/2/24/s1, Table S1: Summary of Test Phase stimuli for one counterbalancing version, Table S2: Output of a mixed effects model for total production growth in relation to Test Phase response Polarity, Table S3: Output of a mixed effects model for total production growth in relation to mispronunciation sensitivity.

## References

[1] Jusczyk P.W., Aslin R.N. (1995). Infants’ detection of the sound patterns of words in fluent speech. Cogn. Psychol..

[2] Männel C., Friederici A.D. (2013). Accentuate or repeat? Brain signatures of developmental periods in infant word recognition. Cortex.

[3] Bosch L., Figueras M., Teixido M., Ramon-Casas M. (2013). Rapid gains in segmenting fluent speech when words match the rhythmic unit: Evidence from infants acquiring syllable-timed languages. Front. Psychol..

[4] Kooijman V., Hagoort P., Cutler A. (2005). Electrophysiological evidence for prelinguistic infants’ word recognition in continuous speech. Cogn. Brain Res..

[5] Minagawa Y., Hakuno Y., Kobayashi A., Naoi N., Kojima S. (2017). Infant word segmentation recruits the cerebral network of phonological short-term memory. Brain Lang..

[6] Nishibayashi L.-L., Goyet L., Nazzi T. (2015). Early speech segmentation in French-learning infants: Monosyllabic words versus embedded syllables. Lang. Speech.

[7] Goyet L., de Schonen S., Nazzi T. (2010). Words and syllables in fluent speech segmentation by French-learning infants: An ERP study. Brain Res..

[8] Nazzi T., Mersad K., Sundara M., Iakimova G., Polka L. (2014). Early word segmentation in infants acquiring Parisian French: Task-dependent and dialect-specific aspects. J. Child Lang..

[9] Newman R.S., Ratner N.B., Jusczyk A.M., Jusczyk P.W., Dow K.A. (2006). Infants’ early ability to segment the conversational speech signal predicts later language development: A retrospective analysis. Dev. Psychol..

[10] Kooijman V., Junge C., Johnson E.K., Hagoort P., Cutler A. (2013). Predictive brain signals of linguistic development. Front. Psychol..

[11] Junge C., Kooijman V., Hagoort P., Cutler A. (2012). Rapid recognition at 10 months as a predictor of language development. Dev. Sci..

[12] Kuhl P.K. (2004). Early language acquisition: Cracking the speech code. Nat. Rev. Neurosci..

[13] Nishibayashi L.-L., Nazzi T. (2016). Vowels, then consonants : Early bias switch in recognizing segmented word forms. Cognition.

[14] Bouchon C., Floccia C., Fux T., Adda-Decker M., Nazzi T. (2015). Call me Alix, not Elix: Vowels are more important than consonants in own-name recognition at 5 months. Dev. Sci..

[15] Nazzi T., Poltrock S., Von Holzen K. (2016). The developmental origins of the consonant bias in lexical processing. Curr. Dir. Psychol. Sci..

[16] Nespor M., Peña M., Mehler J. (2003). On the different roles of vowels and consonants in speech processing and language acquisition. Lingue Linguaggio.

[17] Kooijman V., Hagoort P., Cutler A. (2009). Prosodic structure in early word segmentation: ERP evidence from Dutch ten-month-olds. Infancy.

[18] Junge C., Cutler A., Hagoort P. (2014). Successful word recognition by 10-month-olds given continuous speech both at initial exposure and test. Infancy.

[19] Tsao F.-M., Liu H.-M., Kuhl P.K. (2004). Speech perception in infancy predicts language development in the second year of life: A longitudinal study. Child Dev..

[20] Benedict H. (1979). Early lexical development: comprehension and production. J. Child Lang..

[21] Lucariello J. (1987). Concept formation and its relation to word learning and use in the second year. J. Child Lang..

[22] Bates E., Dale P.S., Thal D., Fletcher P., MacWhinney B. (1995). Individual differences and their implications for theories of language development. Handbook of Child Language.

[23] Mirman D. (2014). Growth Curve Analysis and Visualization Using R.

[24] Mirman D., Dixon J.A., Magnuson J.S. (2008). Statistical and computational models of the visual world paradigm: Growth curves and individual differences. J. Mem. Lang..

[25] Hochmann J.R., Benavides-Varela S., Nespor M., Mehler J., Flo A. (2017). Bias for vocalic over consonantal information in 6-month-olds. Infancy.

[26] Hochmann J.R., Benavides-Varela S., Nespor M., Mehler J. (2011). Consonants and vowels: Different roles in early language acquisition. Dev. Sci..

[27] Poltrock S., Nazzi T. (2015). Consonant/vowel asymmetry in early word form recognition. J. Exp. Child Psychol..

[28] Keidel J.L., Jenison R.L., Kluender K.R., Seidenberg M.S. (2007). Does grammar constrain statistical learning?. Psychol. Sci..

[29] Floccia C., Nazzi T., Luche C.D., Poltrock S., Goslin J. (2014). English-learning one-to two-year-olds do not show a consonant bias in word learning. J. Child Lang..

[30] Koerner T., Zhang Y., Nelson P.B., Wang B., Zou H. (2016). Neural indices of phonemic discrimination and sentence-level speech intelligibility in quiet and noise: A mismatch negativity study. Hear. Res..

[31] Koerner T., Zhang Y., Nelson P.B., Wang B., Zou H. (2017). Neural indices of phonemic discrimination and sentence-level speech intelligibility in quiet and noise: A P3 study. Hear. Res..

[32] Carreiras M., Vergara M., Perea M. (2007). ERP correlates of transposed-letter similarity effects: Are consonants processed differently from vowels?. Neurosci. Lett..

[33] Carreiras M., Duñabeitia J.A., Molinaro N. (2009). Consonants and vowels contribute differently to visual word recognition: ERPs of relative position priming. Cereb. Cortex.

[34] Carreiras M., Gillon-Dowens M., Vergara M., Perea M. (2008). Are vowels and consonants processed differently? Event-related potential evidence with a delayed letter paradigm. J. Cogn. Neurosci..

[35] Mani N., Mills D.L., Plunkett K. (2012). Vowels in early words: An event-related potential study. Dev. Sci..

[36] Duta M.D., Styles S.J., Plunkett K. (2012). ERP correlates of unexpected word forms in a picture-word study of infants and adults. Dev. Cogn. Neurosci..

[37] Mills D.L., Prat C., Zangl R., Stager C.L., Neville H.J., Werker J.F. (2004). Language experience and the organization of brain activity to phonetically similar words: ERP evidence from 14-and 20-month-olds. J. Cogn. Neurosci..

[38] New B., Pallier C., Brysbaert M., Ferrand L. (2004). Lexique 2: A new French lexical database. Behav. Res. Methods Instrum. Comput..

[39] Kern S. (2003). Le compte-rendu parental au service de l’évaluation de la production lexicale des enfants français entre 16 et 30 mois. Glossa.

[40] Goldman J.-P. EasyAlign: An automatic phonetic alignment tool under Praat. Proceedings of the 12th Annual Conference of the International Speech Communication Association InterSpeech.

[41] Boersma P., Weenink D. Praat: Doing Phonetics by Computer (Version 5.4.22). http://www.fon.hum.uva.nl/praat/.

[42] (2012). E-Prime.

[43] (2014). EGI Version.

[44] Delorme A., Makeig S. (2004). EEGLAB: An open sorce toolbox for analysis of single-trail EEG dynamics including independent component anlaysis. J. Neurosci. Methods.

[45] Lopez-Calderon J., Luck S.J. (2014). ERPLAB: An open-source toolbox for the analysis of event-related potentials. Front. Hum. Neurosci..

[46] Alschuler D.M., Tenke C.E., Bruder G.E., Kayser J. (2014). Identifying electrode bridging from electrical distance distributions: A survey of publicly-available EEG data using a new method. Clin. Neurophysiol..

[47] DeBoer T., Scott L., Nelson C.A., De Haan M. (2006). Methods for acquiring and analyzing infant event-related potentials. Introduction to Infant EEG and Event-Related Potentials.

[48] Wickham H. (2009). Ggplot2: Elegant Graphics for Data Analysis.

[49] Arcara G., Petrova A. Erpr: Event-Related Potentials (ERP) Analysis, Graphics and Utility Functions. https://cran.r-project.org/web/packages/erpR/index.html.

[50] Lawrence M.A. Ez: Easy Analysis and Visualization of Factorial Experiments. https://cran.r-project.org/web/packages/ez/index.html.

[51] Bloom L. (1973). One Word at a Time: The Use of Single Word Utterances Before Syntax.

[52] McMurray B. (2007). Defusing the childhood vocabulary explosion. Science.

[53] Swingley D. (2005). 11-month-olds’ knowledge of how familiar words sound. Dev. Sci..

[54] Mayor J., Plunkett K. (2014). Infant word recognition: Insights from TRACE simulations. J. Mem. Lang..

[55] Nazzi T., Bertoncini J. (2009). Phonetic specificity in early lexical acquisition: New evidence from consonants in coda positions. Lang. Speech.

[56] Soares A.P., Perea M., Comesaña M. (2014). Tracking the emergence of the consonant bias in visual-word recognition: Evidence with developing readers. PLoS ONE.

[57] Lee H.W., Rayner K., Pollatsek A. (2002). The processing of consonants and vowels in reading: Evidence from the fast priming paradigm. Psychon. Bull. Rev..

[58] Bonatti L.L., Peña M., Nespor M., Mehler J. (2005). Linguistic constraints on statistical computations: The role of consonants and vowels in continuous speech processing. Psychol. Sci..

[59] New B., Araujo V., Nazzi T. (2008). Differential processing of consonants and vowels in reading. Psychol. Sci..

[60] Cutler A., Sebastian-Gallés N., Soler-Vilageliu O., van Ooijen B. (2000). Constraints of vowels and consonants on lexical selection: Cross-linguistic comparisons. Mem. Cogn..

