# Supplementary material for: Consonant and Vowel Processing in Word Form Segmentation: An Infant ERP Study
Source: Brain Sci. 2018 Jan 31;8(2):24. doi: 10.3390/brainsci8020024 (PMC5836043; doi:10.3390/brainsci8020024)
Supplement: Supplementary file 1 [file brainsci-08-00024-s001.zip › Table_S1.docx]

**Table S1.** Summary of Test Phase stimuli for one counterbalancing version. Words are organized by mispronunciation type (onset consonant, medial vowel, coda consonant) and feature changed (consonants: voicing, place, manner; vowels: height, place, roundness). IPA (International Phonetic Alphabet) transcriptions are given in parenthesis.

| Mispronunciation Type | Feature changed | Target | Mispronunciation | Control |
| --- | --- | --- | --- | --- |
| Onset consonant | voicing | cave (/kav/) | gave (/gav/) | reg (/rɛg/) |
|  |  | pic (/pik/) | bique (/bik/) | sève (/sɛv/) |
|  |  | dôme (/dom/) | tome (/tom/) | gigue (/ʒig/) |
|  |  | schoute (/ʃut/) | joute (/ʒut/) | terre (/tɛr/) |
|  |  | jaffe (/ʒaf/) | chaff (/ʃaf/) | tube (/tyb/) |
|  |  | quiche (/kiʃ/) | guiche (/giʃ/) | scène (/sɛn/) |
|  |  | vis (/vis/) | fil (/fis/) | taule (/tol/) |
|  | place | lyse (/liz/) | rise (/riz/) | tour (/tur/) |
|  |  | dalle (/dal/) | gale (/gal/) | tare (/tar/) |
|  |  | fil (/fil/) | cil (/sil/) | dot (/dɔt/) |
|  |  | lobe (/lɔb/) | robe (/rɔb/) | bar (/bar/) |
|  |  | salle (/sal/) | châles (/ʃal/) | digue (/dig/) |
|  |  | couche (/kuʃ/) | touche (/tuʃ/) | bêche (/bɛʃ/) |
|  |  | pige (/piʒ/) | tige (/tiʒ/) | maque (/mak/) |
|  | manner | toque (/tɔk/) | soc (/sɔk/) | baile (/bɛl/) |
|  |  | bal (/bal/) | malle (/bal/) | schème (/ʃɛm/) |
|  |  | tir (/tir/) | cire (/sir/) | gamme (/gam/) |
|  |  | boule (/bul/) | moule (/mul/) | toge (/tɔʒ/) |
|  |  | louve (/luv/) | douve (/duv/) | gon (/gon/) |
|  |  | dîme (/dim/) | lime (/lim/) | batte (/bat/) |
|  |  | baisse (/bɛs/) | messe (/mɛs/) | folle (/fɔl/) |
| medial vowel | height | guette (/gɛt/) | gatte (/gat/) | fugue (/fyg/) |
|  |  | faine (/fɛn/) | fane (/fan/) | puce (/pys/) |
|  |  | site (/sit/) | set (/sɛt/) | loute (/lut/) |
|  |  | pesse (/pɛs/) | passe (/pas/) | coeur (/kør/) |
|  |  | chic (/ʃik/) | chèque (/ʃɛk/) | leurre (/lør/) |
|  |  | bauge (/boʒ/) | bouge (/buʒ/) | gueule (/gøl/) |
|  |  | chum (/ʃum/) | chaume (/ʃom/) | peuhl (/pøl/) |
|  | place | môle (/mol/) | meule (/møl/) | bile (/bil/) |
|  |  | gut (/gyt/) | goutte (/gut/) | pente (/pãt/) |
|  |  | pull (/pyl/) | poule (/pul/) | ronce (/rɔ̃s/) |
|  |  | souk (/suk/) | suc (/syk/) | gîte (/ʒit/) |
|  |  | port (/pɔr/) | peur (/pør/) | bande (/bãd/) |
|  |  | feurre (/før/) | fort (/fɔr/) | dire (/dir/) |
|  |  | bore (/bɔr/) | beurre (/bør/) | pire (/pir/) |
|  | roundness | panse (/pãs/) | ponce (/pɔ̃s/) | chef (/ʃɛf/) |
|  |  | fonte (/fɔ̃t/) | fente (/fãt/) | mise (/miz/) |
|  |  | tong (/tɔ̃g/) | tangue (/tãg/) | gel (/ʒɛl/) |
|  |  | bise (/biz/) | buse (/byz/) | datte (/dat/) |
|  |  | tic (/tik/) | tuque (/tyk/) | voûtes (/vut/) |
|  |  | rime (/rim/) | rhume (/rym/) | saule (/sol/) |
|  |  | seul (/søl/) | sel (/sɛl/) | rôle (/rol/) |
| coda consonant | voicing | lynch (/l~ɛʃ/) | linge (/l~ɛʒ/) | khat (/kat/) |
|  |  | mage (/maʒ/) | mâche (/maʃ/) | jour (/ʒur/) |
|  |  | bogue (/bɔg/) | boc (/bɔk/) | chose (/ʃoz/) |
|  |  | taube (/tob/) | taupe (/top/) | mouche (/muʃ/) |
|  |  | vif (/vif/) | vive (/viv/) | nappe (/nap/) |
|  |  | gache (/gaʃ/) | gage (/gaʒ/) | ronde (/rɔ̃d/) |
|  |  | bègue (/bɛg/) | bec (/bɛk/) | four (/fur/) |
|  | place | phot (/fɔt/) | phoque (/fɔk/) | vide (/vid/) |
|  |  | fer (/fɛr/) | fêle (/fɛl/) | race (/ras/) |
|  |  | pal (/pal/) | par (/par/) | lente (/lãt/) |
|  |  | riche (/riʃ/) | riss (/ris/) | rade (/rad/) |
|  |  | rouf (/ruf/) | rousse (/rus/) | zen (/zen/) |
|  |  | cob (/kɔb/) | code (/kɔd/) | mite (/mit/) |
|  |  | tasse (/tas/) | tache (/taʃ/) | gade (/gad/) |
|  | manner | coude (/kud/) | coule (/kul/) | rab (/rab/) |
|  |  | cote (/kot/) | cosse (/kos/) | cage (/kaʒ/) |
|  |  | quête (/kɛt/) | caisse (/kɛs/) | fic (/fik/) |
|  |  | bonne (/bɔn/) | bol (/bɔl/) | latte (/lat/) |
|  |  | mine (/min/) | mil (/mil/) | biche (/biʃ/) |
|  |  | mat (/mat/) | masse (/mas/) | dogue (/dɔg/) |
|  |  | cause (/koz/) | cône (/kon/) | soute (/sut/) |
|  |  |  |  |  |
