# Supplementary material for: Consonant and Vowel Processing in Word Form Segmentation: An Infant ERP Study
Source: Brain Sci. 2018 Jan 31;8(2):24. doi: 10.3390/brainsci8020024 (PMC5836043; doi:10.3390/brainsci8020024)
Supplement: Supplementary file 1 [file brainsci-08-00024-s001.zip › Table_S2.docx]

**Table S2.** Output of a mixed effects model for total production growth in relation to Test Phase response Polarity. Model construction: Production.z_score ~ (Linear_time + Quadratic_time + Cubic_time) * Polarity + (Linear_time + Quadratic_time | Subject).

| **Fixed Effects** | **Estimate** | **SE** | **df** | **t value** | **Pr(>\|t\|)** |  |
| --- | --- | --- | --- | --- | --- | --- |
| (Intercept) | 0.17619 | 0.07484 | 29.96 | 2.354 | 0.0253 | *^1^ |
| Linear_time | 1.64485 | 0.19277 | 28.86 | 8.533 | 2.21E-09 | *** |
| Quadratic_time | 1.10588 | 0.13087 | 30.47 | 8.45 | 1.75E-09 | *** |
| Cubic_time | 0.44059 | 0.03577 | 79.19 | 12.318 | < 2e-16 | *** |
| PolarityPos | -0.25044 | 0.11738 | 29.9 | -2.134 | 0.0412 | * |
| Linear_time:PolarityPos | -0.61993 | 0.30226 | 28.89 | -2.051 | 0.0494 | * |
| Quadratic_time:PolarityPos | -0.40072 | 0.20522 | 30.37 | -1.953 | 0.0601 | . |
| Cubic_time:PolarityPos | -0.14234 | 0.05493 | 78.88 | -2.591 | 0.0114 | * |

^1^ Signif. codes: 0 ‘***’ 0.001 ‘**’ 0.01 ‘*’ 0.05 ‘.’ 0.1 ‘ ’ 1.
