# Supplementary material for: Consonant and Vowel Processing in Word Form Segmentation: An Infant ERP Study
Source: Brain Sci. 2018 Jan 31;8(2):24. doi: 10.3390/brainsci8020024 (PMC5836043; doi:10.3390/brainsci8020024)
Supplement: Supplementary file 1 [file brainsci-08-00024-s001.zip › Table_S3.docx]

**Table S3.** Output of a mixed effects model for total production growth in relation to a Bias for consonants or vowels. Model Construction: Production.z_score ~ (Linear_time+Quadratic_time+Cubic_time) *Misp_Sensitivity + (Linear_time+Quadratic_time | Subject).

| **Fixed Effects** | **Estimate** | **SE** | **df** | **t value** | **Pr(>\|t\|)** |  |
| --- | --- | --- | --- | --- | --- | --- |
| (Intercept) | 0.08 | 0.06 | 30.27 | 1.36 | 0.19 |  |
| Linear_time | 1.41 | 0.15 | 29.58 | 9.43 | 2.01e-10 | *** |
| Quadratic_time | 0.95 | 0.10 | 30.66 | 9.57 | 1.02e-10 | *** |
| Cubic_time | 0.38 | 0.01 | 255.20 | 26.73 | 2.00E-16 | *** |
| Misp_Sensitivity | 0.00 | 0.00 | 255.80 | 0.16 | 0.87 |  |
| Linear_time: Misp_Sensitivity | 0.00 | 0.01 | 247.50 | -0.29 | 0.77 |  |
| Quadratic_time: Misp_Sensitivity | 0.00 | 0.01 | 251.20 | -0.56 | 0.58 |  |
| Cubic_time: Misp_Sensitivity | -0.03 | 0.01 | 252.90 | -4.09 | 5.76e-05 | *** |

^1^ Signif. codes: 0 ‘***’ 0.001 ‘**’ 0.01 ‘*’ 0.05 ‘.’ 0.1 ‘ ’ 1.
